# Oxidation of Methionine 77 in Calmodulin Alters Mouse Growth and Behavior

**DOI:** 10.3390/antiox7100140

**Published:** 2018-10-13

**Authors:** Méry Marimoutou, Danielle A. Springer, Chengyu Liu, Geumsoo Kim, Rodney L. Levine

**Affiliations:** 1 Laboratory of Biochemistry, National Heart, Lung, and Blood Institute, Bethesda, MD 20892-8012, USA; mery.marimoutou@nih.gov (M.M.); kimg@nhlbi.nih.gov (G.K.); 2 Murine Phenotyping Core, National Heart, Lung, and Blood Institute, Bethesda, MD 20892-5570, USA; ds628k@nih.gov; 3 Transgenic Core, National Heart, Lung, and Blood Institute, Bethesda, MD 20892-8403, USA; liuch@nhlbi.nih.gov

**Keywords:** calmodulin, methionine sulfoxide reductase A, methionine sulfoxide, methionine, reversible covalent modification

## Abstract

Methionine 77 in calmodulin can be stereospecifically oxidized to methionine sulfoxide by mammalian methionine sulfoxide reductase A. Whether this has in vivo significance is unknown. We therefore created a mutant mouse in which wild type calmodulin-1 was replaced by a calmodulin containing a mimic of methionine sulfoxide at residue 77. Total calmodulin levels were unchanged in the homozygous M77Q mutant, which is viable and fertile. No differences were observed on learning tests, including the Morris water maze and associative learning. Cardiac stress test results were also the same for mutant and wild type mice. However, young male and female mice were 20% smaller than wild type mice, although food intake was normal for their weight. Young M77Q mice were notably more active and exploratory than wild type mice. This behavior difference was objectively documented on the treadmill and open field tests. The mutant mice ran 20% longer on the treadmill than controls and in the open field test, the mutant mice explored more than controls and exhibited reduced anxiety. These phenotypic differences bore a similarity to those observed in mice lacking calcium/calmodulin kinase IIα (CaMKIIα). We then showed that MetO77 calmodulin was less effective in activating CaMKIIα than wild type calmodulin. Thus, characterization of the phenotype of a mouse expressing a constitutively active mimic of calmodulin led to the identification of the first calmodulin target that can be differentially regulated by the oxidation state of Met77. We conclude that reversible oxidation of methionine 77 in calmodulin by MSRA has the potential to regulate cellular function.

## 1. Introduction

Except for its role in protein initiation, methionine had typically been viewed as a generic hydrophobic amino acid but investigations from many laboratories have changed this view [1,2,3,4,5,6,7,8]. Methionine in proteins functions in antioxidant defense, protein structure and redox sensing/regulation. Reversible covalent modification of proteins, such as phosphorylation and dephosphorylation, has long been appreciated to be a key mechanism of cellular regulation. In the same way, cyclic oxidation and reduction of methionine has the potential to function as a regulatory switch. Such a regulatory function has been established for methionine in actin. The flavin-containing NADPH-dependent oxidoreductase MICAL (Microtubule Associated Monooxygenase, Calponin And LIM Domain Containing)mediates the stereospecific oxidation of Met44 in F-actin, leading to its depolymerization [6]. The cytosolic methionine sulfoxide reductase B1 (MSRB1) reduces methionine sulfoxide 44 (MetO44) back to Met, restoring its ability to polymerize [4,5]. The reactions are specific for the R-epimer of MetO. Methionine sulfoxide reductase A (MSRA) is stereospecific for the S epimer and it is a bifunctional enzyme, capable of stereospecifically oxidizing Met and reducing MetO residues [9].

Calmodulin is a substrate for MSRA with an intriguing specificity. There are 9 Met residues in calmodulin, each of which can be oxidized by low molecular weight agents such as hydrogen peroxide or hypochlorous acid. However, MSRA oxidizes only Met77 to S-MetO77 and this can be completely reversed when MSRA operates in the reductase direction [10]. There are hundreds of known targets of calmodulin [11] but to date, regulation by methionine oxidation and reduction in vivo by MSRA has only been implicated in the ripening of bananas [12].

**Figure 1:**
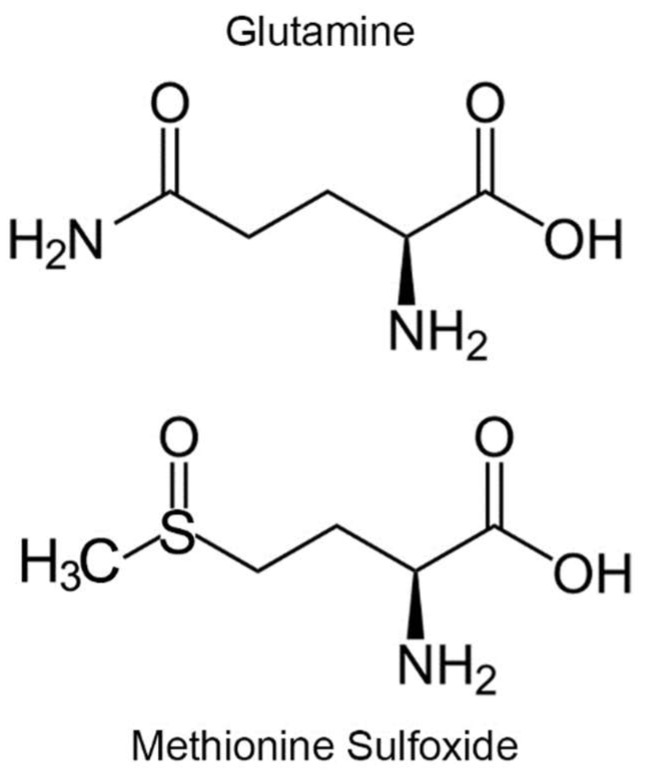
Glutamine is an analogue of methionine sulfoxide.

The mammalian genome contains 3 genes encoding calmodulin, all yielding the identical protein sequence but differing in their 5′ promotor and 5′- and 3′-untranslational regions [13]. Thus, expression of the 3 genes varies from tissue to tissue. The mRNA for calmodulin-1 is highly expressed in brain, testis, intestine and muscle. Expression of calmodulin-2 is very high in brain and testis and well expressed in placenta, lung, thymus, muscle and intestine. Expression of calmodulin-3 is high in brain and testis. In this communication, we report the results of two approaches towards our goal of identifying mammalian targets that are regulated by redox modulation of Met77 in calmodulin. In the first approach, we utilized a micro protein array to identify candidate binding partners of calmodulin. In the second approach, we created a mutant mouse in which Met77 was mutated to glutamine on both copies of the calmodulin-1 gene (*Calm1*) to create a model of constitutive oxidation of calmodulin-1. Glutamine is a structural analogue of MetO (Figure 1), the hydrophobicities of glutamine and MetO are the same and substitution of Met by glutamine has been demonstrated to mimic MetO [12,14,15,16]. However, glutamine is not a perfect mimic, as glutamines can pack well into an alpha helix while MetO is incompatible [17].

## 2. Materials and Methods

### 2.1. Recombinant Calmodulins and Assay of Calcium/Calmodulin Dependent Protein Kinase IIα (CaMKIIα)

Wild type and M77Q calmodulins were produced recombinantly to serve as probes of the protein array. MetO77 calmodulin was produced by incubation of wild-type calmodulin with myristoylated mouse MSRA as described [10] and was >99% MetO form after incubation. M77Q calmodulin DNA (pET17b) was obtained using a QuikChange II Site-Directed Mutagenesis Kit (Agilent 200523, Santa Clara, CA, USA). Primers were 5′-ACCATGATGGCCAGAAAGCAGAAGGACACAGACAGTGAG-3′ and 5′-CTCACTGTC TGTGTCCTTCTGCTTTCTGGCCATCATGGT-3′ with human calmodulin (pET17b) as the DNA template. Proteins were expressed in *E. coli* BL21(DE3) cells that were grown at 37° in lysogeny broth (LB) medium containing 100 ug/mL ampicillin. When the optical density at 600 nm reached 0.5, 0.1 mM isopropyl 1-thio-d-galactopyranoside was added to induce protein expression. Three hours later, cells were harvested by centrifugation at 4° for 25 min at 4000× *g*. The pellet weighed 8 g. Pellets were resuspended in 50 mM Na_2_HPO_4_, pH 7.4, with 1 × Protease Inhibitor Mixture I (Millipore 539134, Burlington, MA, USA), 1 mM phenylmethanesulfonyl chloride, 1 mM Ethylenediaminetetraacetic acid (EDTA) and 1% Triton X-100 (Sigma T9284, St. Louis, MO, USA) and then disrupted by French press at 20,000 psi with a cell fitted with a 1 inch piston (Thermo Spectronic, Rochester, NY, USA). The homogenates were centrifuged at 4° for 20 min at 25,000× *g*, which was also the case for all following centrifugations. The supernatant was then made 1% *w*/*v* in streptomycin (Sigma S6501-100G) to precipitate nucleic acids. After rocking 15 min at 4°, the sample was centrifuged. The supernatant was then brought to 65% saturation ammonium sulfate by addition of solid ammonium sulfate (Sigma A2989-1KG), rocked for 15 min at 4° and centrifuged. The pellet was saved and the supernatant was brought to 90% saturation in ammonium sulfate. The solution was rocked again for 15 min at 4° and was then centrifuged. Pellets were redissolved in and an aliquot analyzed by sodium dodecyl sulfate polyacrylamide gel electrophoresis (SDS PAGE) gel and a Coomassie staining. Both wild type calmodulin and M77Q calmodulin were mostly present in 65–90% ammonium sulfate pellet. These pellets were resuspended in buffer A (Na_2_HPO_4_ 50 mM, diethylenetriaminepentaacetic acid (DTPA) 1 mM, pH 7.9) while the supernatants were dialyzed 3 times against buffer A.

The redissolved pellets from the 65–90% ammonium sulfate cuts of calmodulin and M77Q calmodulin were further purified, with the first chromatographic step being anion exchange chromatography (TosoHass DEAE-5PW, 21.5 mm ID, 15 cm length, 13 µm particle size). Proteins were eluted by a gradient of buffer B (Na_2_HPO_4_ 50 mM, DTPA 1 mM, 1 M NaCl, pH 7.9) developed at 2.5%/min at a flow of 3 mL/min. After dialysis against buffer A, solid ammonium sulfate was added to a final concentration of 1 M. Fractions were then subjected to hydrophobic interaction chromatography (TosoHass Phenyl-5PW, 21.5 mm ID, 15 cm length, 13 µm particle size), initially pumping buffer C (Na_2_HPO_4_ 50 mM, DTPA 1 mM, ammonium sulfate 1 M, pH 7.9) at 3 mL/min. Proteins were eluted by a gradient of buffer A developed at 2.3%/min.

The expected mass of the purified proteins was confirmed by HPLC- time of flight mass spectroscopy [1]. They were 16,706.9 Da for wild type calmodulin (99% purity) with a calculated mass of 16,706.4 Da and 16,703.8 Da for M77Q calmodulin (70% purity) with a calculated mass of 16,703.4 Da. The amount of both calmodulins was measured by the Bradford method [18] using Bio-Rad protein assay reagent and bovine serum albumin as a standard. The yield was 30 mg of wild-type calmodulin and 7.5 mg of the M77Q mutant.

To quantitate tissue calmodulin, 50 mg of heart or muscle were minced and homogenized in 500 µL RIPA buffer (Sigma R0278) with 1 mM phenylmethanesulfonyl chloride and 1 × Millipore Protease Inhibitor Mixture. Brain tissues were minced and then homogenized with a pestle. Muscle and heart tissues were homogenized using a Polytron homogenizer for 20 s. Samples containing 10 µg protein were reduced in SDS-PAGE buffer (Life Technologies LC2676, Carlsbad, CA, USA) containing 0.5% mercaptoethanol by heating at 95° for 5 min. Gel electrophoresis was performed on 10–20% Tris/glycine gels (15-well, 1.5 mm; Invitrogen, XP10205BOX). A constant output power of 180 V was applied at room temperature for 90 min. The proteins were then transferred to a nitrocellulose membrane (Biorad, 704158, Hercules, CA, USA). The membrane was incubated with a 1:1000 dilution of anti-calmodulin antibody (Abcam, catalog ab455689, Cambridge, MA, USA) overnight at 4° and then with a 1:5000 dilution of the secondary antibody (Alexa Fluor 680 goat anti-rabbit IgG (Invitrogen A21109, Carlsbad, CA, USA) for 1 h at room temperature. The membrane was washed four times with 0.1% Tween-20 (Sigma P5927) in phosphate-buffered saline (KD Medical RGF-3210). The calmodulin content was quantified by scanning with an Odyssey infrared scanner and associated Image Studio version 3 software (Li-Cor Biosciences, Lincoln, NE, USA). Calmodulin recombinant protein was used as the standard, loading from 0 to 10 ng per lane.

CaMKIIα was assayed at 37° with an incubation time of 10 min. The 10 µL assay solution for CaMKIIα contained 50 mM HEPES (Teknova H0135, 1 M stock, pH 7.5), 10 mM magnesium acetate, 0.5 mM calcium chloride, 0.4 mM ATP, 50 µM syntide-2 (PLARTLSVAGLPGKK; AnaSpec AS-22552), 1 mg/mL bovine serum albumin and calmodulin ranging from 10^−9^ to 10^−6^ M. The assay tubes were pre-incubated for 2 min and then the reaction was started by the addition of 20 ng CaMKIIα (Abcam ab60899). It was stopped by addition of 0.5 µL of 20% trifluoroacetic acid. Phosphorylated syntide-2 was separated from unmodified syntide-2 by HPLC on a Zorbax 300Å StableBond C18 MicroBore column (1.0 × 50 mm, 3.5 µm particle size, Agilent 865630-902) with an Agilent 1200 series high pressure liquid chromatography system equipped with an autosampler set to 4° and a column compartment set to 30°. The initial solvent was water/0.05% trifluoracetic acid and peptides were eluted by a gradient of acetonitrile/0.05% trifluoroacetic acid with a flow rate of 20 µL/min. Acetonitrile was ramped to 10% over 2 min, then to 22% over the next 24 min and the column was washed by ramping to 95% in 4 min. With this program, phosphorylated syntide-2 eluted at 23.7 min and unphosphorylated syntide-2 at 24.4 min. The two forms of the peptide were quantitated from their areas in the chromatogram recorded at 210 nm with a 10 nm bandwidth. The calmodulin concentration curves were fit to an allosteric response and compared with Prism version 7 (GraphPad Software, La Jolla, CA, USA).

### 2.2. Protein Array

Wild-type and M77Q calmodulins were biotinylated with maleimide-PEG2-Biotin (Invitrogen, # 21901) and then dialyzed 50 mM HEPES, 150 mM NaCl and 1 mM DTPA. The expected increase in mass of 525.6 Da was confirmed by HPLC-time of flight mass spectroscopy. The concentrations, ~300 µM were measured after which an aliquot was diluted to 0.10 µM in buffer I (50 mM HEPES, 150 mM NaCl, 5% glycerol, 0.05% TritonX-100, 1% bovine serum albumin, 5 mM MgCl_2_, 0.5 mM dithiothreitol (DTT) and 2 mM CaCl_2_). The solution was incubated with 0.2 µg/mL streptavidin-conjugated Alexa 647 (Invitrogen S32357) in the dark at room temperature for 1 h. Protein arrays (ProtoArray**^®^** Human Protein Microarray v. 5.0, Invitrogen) were incubated with blocking buffer (50 mM HEPES, 150 mM NaCl, 0.1% Tween-20 and 1% bovine serum albumin (BSA)) for 1 h at 4° and then incubated with the biotin-streptavidin complex for 2 h at 4°. Arrays were then washed 3 times with washing buffer II (50 mM HEPES, 150 mM NaCl, 0.1% Tween-20, 1% BSA, 5 mM MgCl_2_ and 0.5 mM dithiothreitol [DTT]). Arrays were scanned with a GenePix 4200AL (Molecular Devices, San Jose, CA, USA) and analyzed with Invitrogen’s ProtoArray Prospector software (Thermo Fisher Scientific, Waltham, MA, USA).

### 2.3. Generation of the Calmodulin M77Q Mouse

All animals were treated humanely in accordance with the Guide for the Care and Use of Laboratory Animals [19]. The study was approved by the Animal Care and Use Committee of the National Heart, Lung and Blood Institute (Study H-120R4 latest approval on 7 November 2017). An ~200 bp genomic DNA sequence of *Calm1* surrounding Met77 was submitted into MIT’s CRISPR design website [20]. Two sgRNAs were selected based on their off-target scores and proximity to the mutation site, one upstream (Cam1-Upstream: CCCAGAGTTCTTGACTATGA) and the other downstream (Calm1-Downstream: CAAACACTCGGAAGGCCTCG) of the Met77. The Calm1-Upstream sgRNA is expected to cut 14 bp upstream of Met77, the targeted mutation site. The Cam1-Downstream sgRNA is expected to cut 34 bp downstream of Met77. 

The sgRNA binding sequences were cloned into a plasmid vector containing a T7 promoter using OriGene’s (Rockville, Maryland) sgRNA cloning service. These plasmids were then used as templates for generating sgRNAs using the MEGAshortscript T7 Kit (ThermoFisher). For each sgRNA, a corresponding oligonucleotide donor (Calm1-Upstream: ACTGTTTCTCTTCTCATTAAAGGCAATGGCACCATTGACTTCCCCGAGTTTCTGACTATGATGGCTAGAAAACAGAAAGACACAGATAGCGAAGAAGAGATCCGCGAGGCCTTCCGAGTG; and Cam1-Downstream: GGCACCATTGACTTCCCAGAGTTCTTGACTATGATGGCTAGAAAACAGAAAGACACAGATAGCGAAGAAGAGATTCGGGAGGCCTTCCGAGTGTTTGACAAGGTAATCTTGCACACTGGCCTT) was purchased from Integrated DNA Technologies (Skokie, Illinois), which contains not only the desired changes for mutating Met77 but also 2–3 silent mutations which do not result in any amino acid changes but can assist in preventing Cas9 from continuingly cutting the DNA after the donor is knocked in. Each sgRNA (50 ng/µL) and its corresponding donor oligonucleotides (100 ng/µL) were co-microinjected with Cas9 mRNA (100 ng/µL from Trilink BioTechnologies, San Diego, CA, USA) into the cytoplasm of zygotes collected from C57BL/6N mice. Injected embryos were cultured in M16 medium in a 37° incubator with 6% CO_2_. When embryos reached the 2-cell stage of development, they were implanted into the oviducts of pseudopregnant surrogate mothers. Offspring born to the foster mothers were genotyped by PCR and DNA sequencing for identifying founders with the desired nucleotide changes. After expansion of the colony, genotyping was carried out by PCR. Briefly, genomic DNA was extracted from tail snips using the REDExtract-N-Amp™ Tissue PCR Kit (Sigma XNAT-100RXN) and amplified by PCR with these primers: (5′-TGCACCTGTAGGTGCTCTGGGCACCGCC-3′, 5′-GACTGCCCCATCTTTGTTCTGTTTGG-3′). DNA sequencing was performed by Macrogen USA.

### 2.4. Treadmill Test

Exercise capacity was tested using a Columbus Instruments rodent treadmill (Model Eco-6M), set at a 10 degree incline. Total exercise time, distance, maximum speed and work were recorded at the time of exhaustion which was defined as the moment the mouse was unable to continue running without repeatedly falling back onto the shock grid at the backend of the treadmill belt. The testing protocol was as follows: 10 min at 10 m/min, then 12 m/min for five minutes. At minute 15 the belt speed was increased to 15 m/min for three minutes and then increased 1.8 m/min every three minutes until the mouse became exhausted.

### 2.5. Dobutamine Cardiac Stress Test

Six-month old mice were lightly anesthetized with isoflurane and placed on a heated platform with ECG leads and a rectal temperature probe to perform echocardiography exams before and after administering constant rate dobutamine infusions. Heart images were acquired using the Vevo2100 ultrasound system (VisualSonics, Toronto, ON, Canada) with a 30 MHz ultrasound probe (VisualSonics, MS-400 transducer). After the baseline-scan, the mice received constant rate infusions of dobutamine (0.625 mg/mL in normal saline containing 5% dextrose), via the tail vein using an infusion syringe pump (Harvard Apparatus, Holliston, MA, USA). The low dose infusion rate was 10 µg/kg/min. After the heart rate reached a steady state as determined by the ECG, the rate was increased to 40 µg/kg/min and the scans repeated.

### 2.6. Spatial Learning Assessment with the Morris Water Maze

A square platform with each side measuring 10 cm was placed in the northwest quadrant of a 6 foot diameter pool and submerged 1 cm below the water line. Non-toxic white tempura paint was added to the water so that the platform was not visible to the mice. Visual clues were placed around the pool and the room during the testing. Mice were acclimated and screened for swimming ability the day before the test. Mice received six sets of four 60 s trials over three consecutive days. The latency time to locate the platform was averaged for each set of four trials. On the last day, a 90 s probe trial was conducted to assess spatial memory. The platform was removed and the time the mice spent in each of the four quadrants of the pool was recorded. The total crossings in the zone that matched the location of the removed platform were also measured. Testing was measured and scored with Anymaze (Stoelting) video tracking system.

### 2.7. Open Field Test

Mice were removed from their home cage and individually placed into a 16″ × 16″ × 16″ Perspex arena viewing chamber where they were allowed to explore for 30 min. Movements were recorded and analyzed with the Anymaze video tracking system. After the test, the mice were returned to their home cage.

### 2.8. Associative Learning Test

On day 1, mice were placed in a 17 cm × 17 cm × 25 cm animal enclosure inside a sound attenuating chamber (Ugo Basile, Gemonio, Italy). The animal enclosure has a grid floor, lights on, clear Plexiglass walls and the scent of vanilla to create a unique environmental context (context 1). Baseline freezing was scored during the first three min in context 1 with the AnyMaze system. During the second three min, animals were exposed to two pairings of fifteen second 4000 Hz tones followed immediately by two 0.85 mA foot shocks of 3 s duration, delivered through the grid floor. 

Twenty four hours after completing context 1, the contextual learning test was conducted. Mice were placed back into the enclosure for three minutes with no auditory or shock stimuli and their freezing was measured. One hour later, mice were placed into a novel chamber (context 2) that was located in a different area of the room. This chamber had a different enclosure geometry and striped walls, with the lights were off, background noise on, a floor that was solid with bedding and no olfactory cue was provided. The auditory tone from day 1 was played in the novel enclosure and freezing time in response to the cue was recorded.

### 2.9. Body Composition

The body composition of mice was analyzed noninvasively at 18 months of age with an EchoMRI™-100H (EchoMRI LLC, Houston, TX, USA). The mouse was placed in a clear plastic tube which was plugged by a plunger with air holes. The plunger was fitted to the mouse and tightened just sufficiently to minimize movement. The tube was then inserted into the instrument to a premeasured depth and measurements were collected. The mouse was then returned to its home cage.

## 3. Results

### 3.1. Protein Array

Oxidation and reduction of Met77 in calmodulin could function as an on-off switch by modulating interaction of the calmodulin with one or more specific targets. We used a human protein array to detect interactions of wild-type or M77Q calmodulin. The arrays are printed on nitrocellulose and have the same dimensions as cDNA arrays used for transcriptomics. They are also scanned in the same instruments as cDNA arrays. The protein array we employed has 9483 human proteins, produced in baculovirus with glutathione-S-transferase (GST) tags [21]. We had earlier used these arrays to demonstrate a protein-protein interaction of methionine sulfoxide reductase A that had not been detected by a number of other techniques [22]. In this study, we interrogated the protein arrays with recombinant wild-type or M77Q calmodulin. Although a number of proteins on the array bound the test proteins, none displayed a difference between the wild-type and M77Q forms.

### 3.2. Calm1 was Mutated in M77Q Mice

Two independent sgRNAs and corresponding donor oligonucleotides were designed and separately microinjected into C57BL/6N zygotes. Ten live offspring were obtained from embryos injected with Calm1-Upstream sgRNA and oligos but none of them carried the desired amino acid changes. However, 10 out 16 mice injected with Calm1-Downstream sgRNA and oligos harbored the desired Met to Gln change. This is a surprisingly high efficiency for an oligonucleotide-mediated knockin, especially considering that the introduced nucleotide changes are 34 bp away from the predicted sgRNA cutting position, because it is generally believed that the efficiency of single strand donor-mediated knockins decreases dramatically if the distance between the mutation site and cutting site is greater than 15–20 bp. We anticipated that the upstream sgRNA is more efficient than the downstream one because it cuts closer to Met77 but the exact opposite was observed here. These observations indicate that there are other factors determining the efficiency of oligonucleotide-mediated knockin.

**Figure 2:**
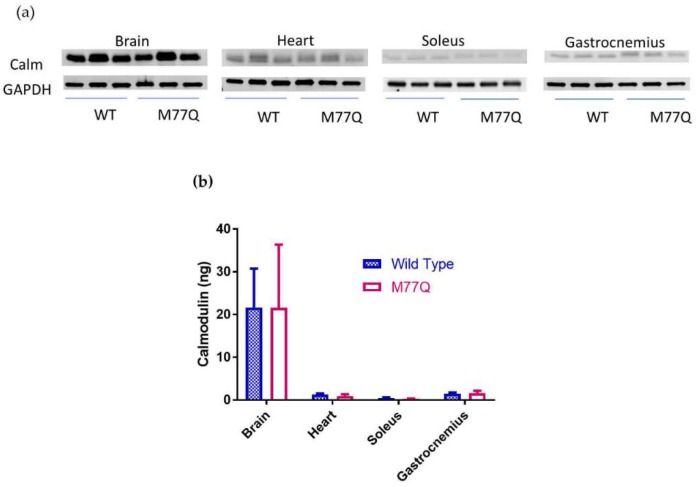
Measurement of total tissue calmodulin in 3 male mice. (**a**) Western blots; (**b**) Quantitation of blots. The abbreviations are Calm, calmodulin and WT, wild type.

DNA analysis of 2 independent founders confirmed that they harbored the desired mutation. These founders were then bred with C57BL/6N wild type mice for expanding the lines, as well as for eliminating possible mosaicism and diluting out off-target effects, if any. Genomic sequencing confirmed that the Met77 codon was changed to Gln77 in both copies of the Calm1 gene. Measurement of calmodulin showed that there was no change in tissue content (Figure 2).

### 3.3. M77Q Mice are Growth Retarded When Younger and Become Fatter When Older

**Figure 3:**
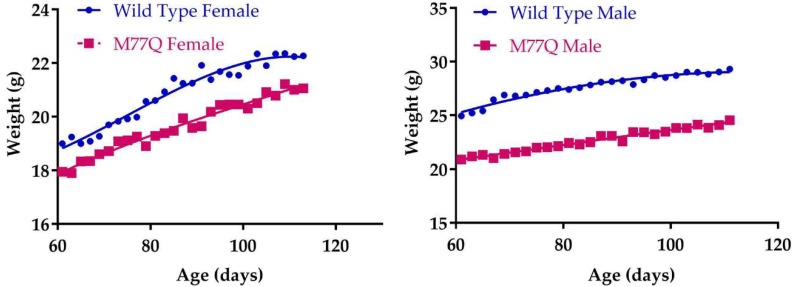
Growth curves. There were 7 mice in each group.

**Figure 4:**
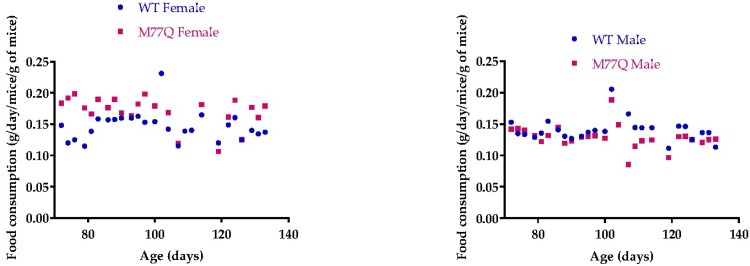
Food consumption. There were 4 mice in each group. The points are the average for the 4 mice. Regression lines were plotted separately for wild-type and M77Q mice but their slopes were not statistically significantly different.

**Figure 5:**
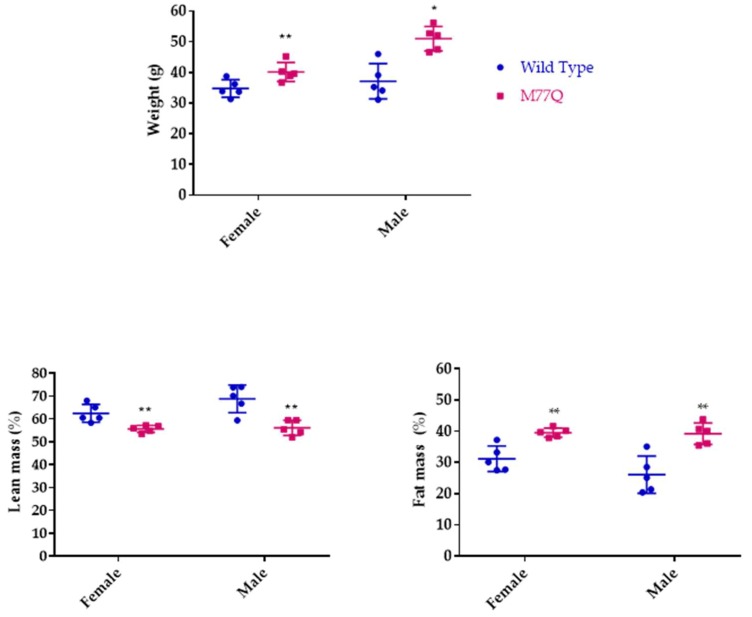
Body composition of mice at 18 months of age. There were 5 mice in each group. (* *p* < 0.05, ** *p* < 0.01 by *t*-test).

M77Q mice were born at term, were viable and could reproduce. However, they were noticeably symmetrically smaller than their wild-type litter mates. We thus followed their growth from 2 to 4 months of age. Both males and females weighed ~20% less than wild-type mice (Figure 3). However, their food intake per gram of body weight was the same as wild-type animals, so that they were constitutively smaller, indicating that the M77Q calmodulin affected growth programs (Figure 4). As the animals aged, we observed the M77Q mice became distinctly fatter than the wild type mice. This was confirmed by MRI determination of the lean and fat masses of the animals (Figure 5).

### 3.4. Cardiac Stress Test and Endurance Running

**Figure 6:**
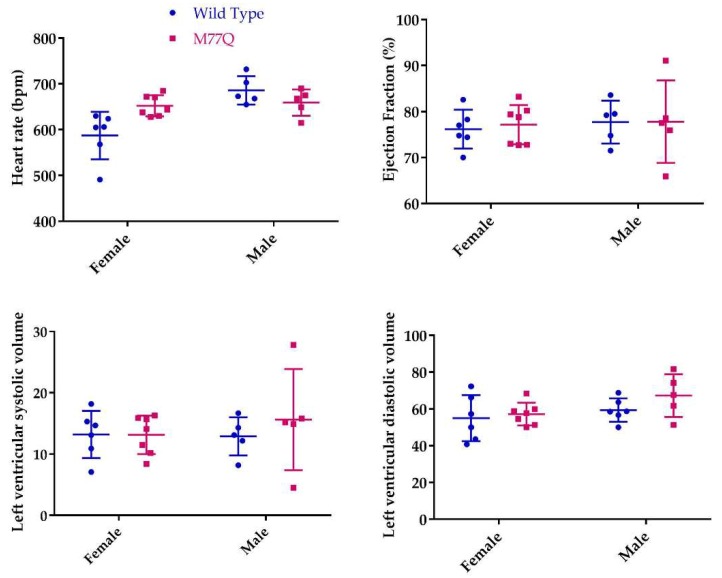
Cardiac parameters during high dose dobutamine stress test at 6 months of age. Except for the female heart rates (*p* = 0.05), none of the comparisons were statistically significantly different.

The relatively high level of expression of Calm1 in heart and skeletal muscle prompted assessment of cardiac function by a pharmacological stress, namely the intravenous infusion of dobutamine to increase cardiac rate and contractility. Except for the female heart rates, there was no difference between the wild-type and M77Q mice in the measured parameters, even at the highest dose of dobutamine (Table 1 and Figure 6).

**Figure 7:**
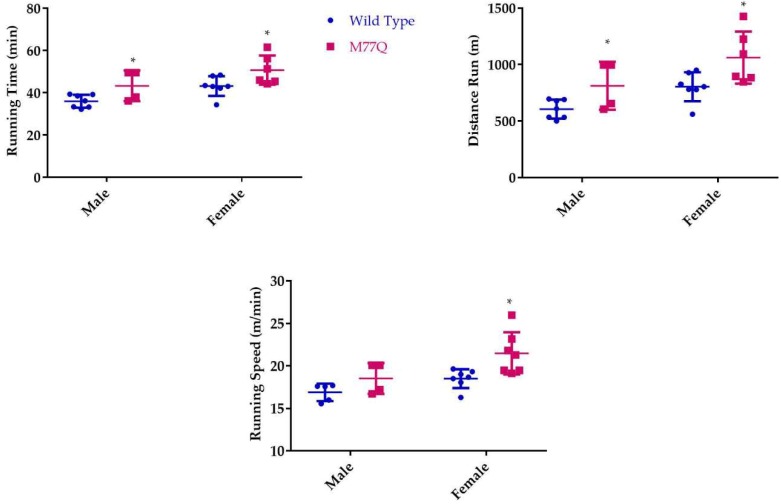
Treadmill test at 6 months of age. We tested 7 wild type and 4 M77Q males and 11 wild type and 8 M77Q females. The results from 1 M77Q were omitted as an outlier because they were greater than 3 standard deviations from the mean of the group. An asterisk marks the M77Q mice that differed significantly from the wild type (* *p* < 0.05 by *t*-test).

**Figure 8:**
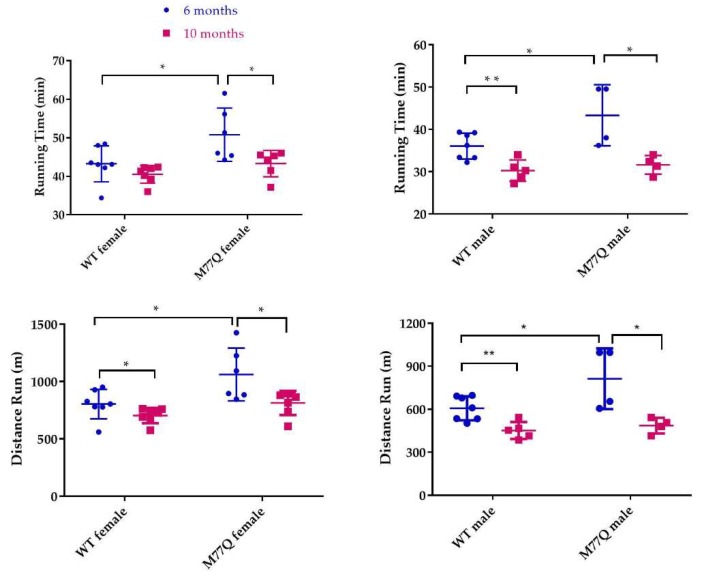
Comparison of treadmill test at 6 and 10 months of age. At 10 months, 5 wild type and 4 M77Q males were tested. Six wild type and 7 M77Q females were tested. * *p* < 0.05, ** *p* < 0.01, by *t*-test.

We observed that young M77Q mice were very active, running in their cages considerably more than their wild-type litter mate controls. This increased activity was evident by 2 months of age. We also noted that the increased activity decreased by 10 months of age. We therefore tested the mice at 6 and 10 months of age in which they run on a treadmill of increasing speed to the point of exhaustion [23]. When tested at 6 months of age, M77Q mice ran 20% longer, with a 30% increase in total distance run (Figure 7). Female M77Q mice ran slightly faster than wild-type, while the speed of running of the males did not reach a statistically significant difference. When the same mice were retested at 10 months of age, wild-type mice exhibited a modest but significant decrease in performance while the M77Q mice had a much greater decline (Figure 8). With these age-related changes, at 10 months of age, there was no difference in performance between wild-type and M77Q mice.

### 3.5. Neurobehavioral Phenotyping

**Figure 9:**
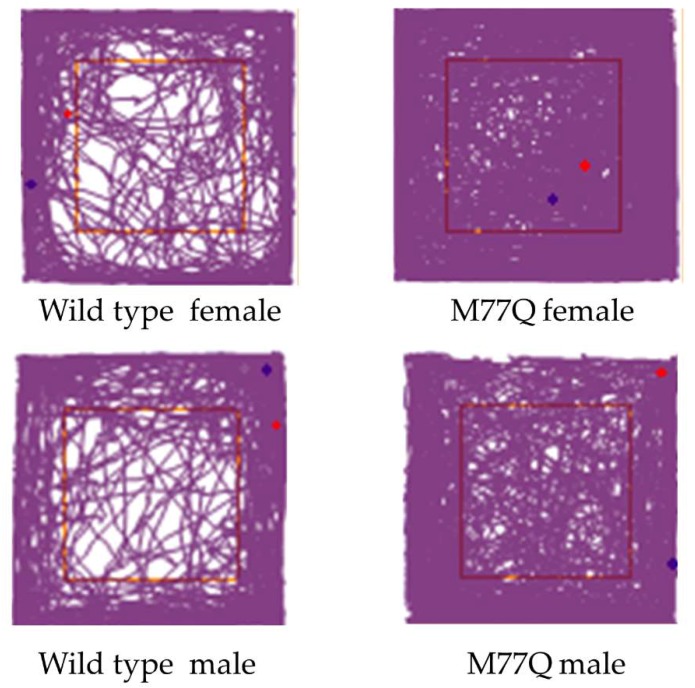
Track plots of mice during the open field test at 6 months of age.

**Figure 10:**
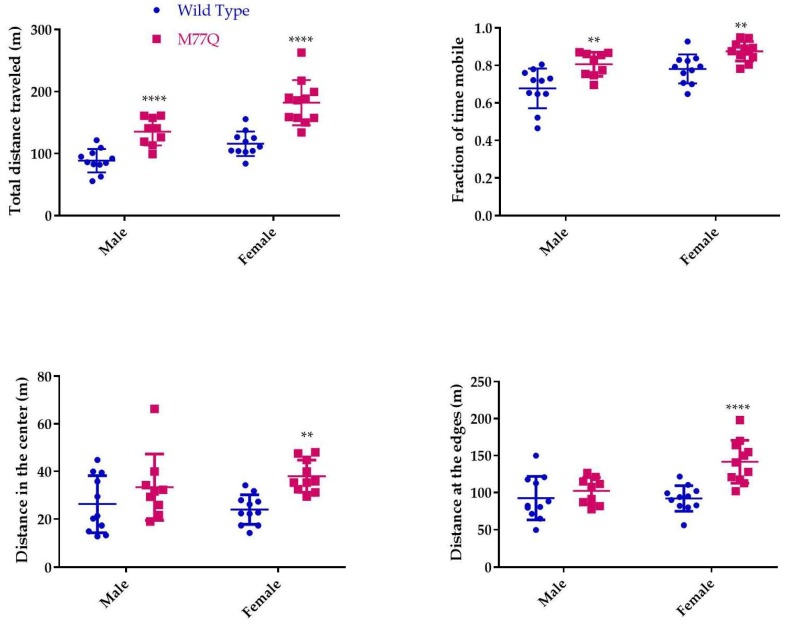
Quantitation of performance on the open field test. We tested 11 wild type and 9 M77Q males and 11 wild type and 10 M77Q females. ** *p* < 0.01, **** *p* < 0.0001 by *t*-test.

The very high level of expression of Calm1 in the brain led us to administer several tests to evaluate behavior, learning and memory, all at 6 months of age. The open field test of spontaneous activity requires normal motor skills and is suited for evaluation of anxiety level and response to a novel environment [24]. In this test, the mouse is placed in a square box for 30 min and movement is recorded by a camera and evaluated by computer. Wild-type mice move more around the periphery, avoiding the central region. Mice that spend more time in the center are considered to have reduced levels of fear of danger from predators. The M77Q mice readily explored the central area while wild-type controls tended to avoid it (Figure 9). The M77Q mice were mobile for a greater fraction of their time in the box and they traveled farther (Figure 10). These objective results are consistent with our subjective impression that the M77Q mice were much more active in their cages than the wild-type mice. The open field test was not repeated at 10 months of age.

**Figure 11:**
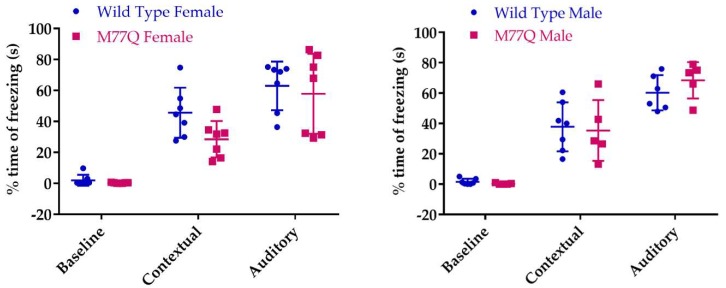
Fraction of time in which the mice freeze during the associative conditioning test. We tested 7 wild type and 5 M77Q males and 7 wild type and 7 M77Q females.

We assessed associative learning with a classical Pavlovian conditioning test [25] in which associative learning is quantitated by the time that the mice freeze their movements. On the first day, they are placed in a box and a sound is delivered via a speaker. Thirty seconds later they receive a mild electrical footshock. The next day they are returned to the identical box to test their contextual memory. On the third day, they are placed in a very different box and the acoustic stimulus is again played in order to evaluate learning from the sound. As expected, freezing time increased with both the contextual and acoustic tests but there was no difference between the wild-type and M77Q mice indicating intact associative learning in the mutant (Figure 11).

**Figure 12:**
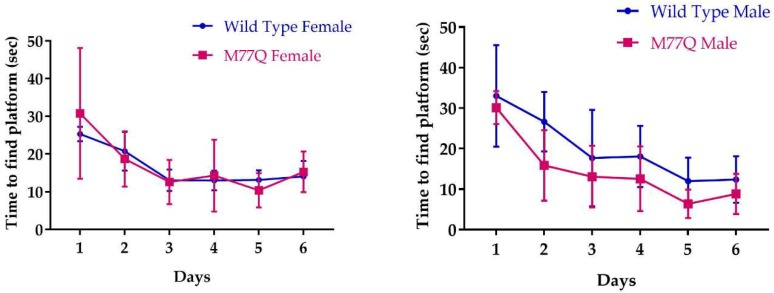
Learning performance on the Morris water maze. We tested 7 wild type and 5 M77Q males and 7 wild type and 7 M77Q females.

We also assessed learning with the Morris water maze in which visual cues guide a mouse to a hidden underwater platform so that they may stand rather than being forced to swim [26]. The test is repeated daily, during which the time for a normal mouse to reach the platform decreases as it learns to follow the visual cues. As in the associative learning test, we did not observe any difference between the wild type and M77Q mice (Figure 12).

### 3.6. Activation of Calcium/Calmodulin Dependent Protein Kinase II

**Figure 13:**
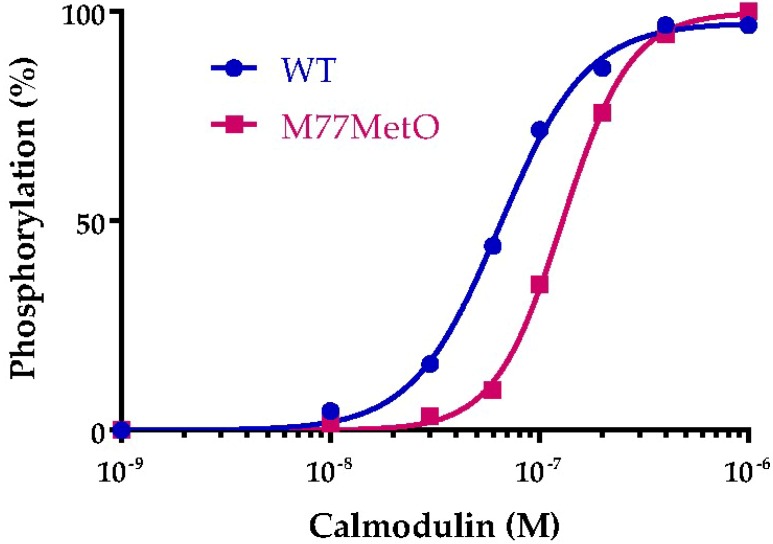
Activation of CaMKIIα by wild type and M77MetO calmodulin. The experiment was performed in 3 separate experiments on different days and the values for each concentration of calmodulin were averaged to generate the plotted points. Curves were fit to an allosteric dose response by Prism.

Mignogna and Viggiano compiled a database of mouse knockouts that displayed altered motor activity [27]. They identified 46 genes that caused increased activity when deleted, two of which are well known calmodulin dependent enzymes: cyclic phosphodiesterase 1 and (CaMKIIα). We previously compared the activation of cyclic phosphodiesterase 1 by wild-type and Met77MetO calmodulins and found them to be identical. With regard to CaMKIIα, mice lacking this kinase are more active in the open field test and spend more time in the center than do control mice [28], as we observed for our mutant mice. From the crystal structure of calmodulin bound to CaMKIIδ, one can see that oxidation of Met77 to MetO could cause a clash with Met308 of CaMKIIδ [29]. We therefore compared activation of CaMKIIα by wild-type and MetO calmodulins (Figure 13). We found that MetO calmodulin is indeed less effective in activating CaMKIIα than the wild type calmodulin (*p* < 0.0001 by extra sum of squares F test).

Half maximal activation required 64 nM wild type calmodulin while half maximal activation with M77MetO calmodulin required 129 nM. The concentration of calcium-bound calmodulin required to activate dependent proteins varies from <10 nM for high-affinity targets, 10–100 nM for intermediate affinity targets and >100 nM for low affinity targets [30]. Thus, the shift from 64 to 129 nM can have substantial effects on calmodulin dependent proteins.

## 4. Discussion and Conclusions

Calmodulin consists of two lobes, one formed by the amino terminal domain and one formed by the carboxy terminal domain [31]. These are joined by a connecting alpha helix, so that the structure resembles a dumbbell. Two calcium ions can be bound in each lobe and methionine residues in each lobe are important in the subsequent interaction of calmodulin with its targets. Met77 is situated very close to the end of the connecting alpha helix. While it is established that MSRA can mediate the reversible oxidation of calmodulin at Met77, to date there are no reports that implicate Met77 in target specificity. Thus, the aim of our study was to identify calmodulin-dependent proteins whose activity or function were modulated by covalent modification of Met77.

To this end, we first employed a human protein array with almost 10,000 proteins as a bioaffinity technique to detect potential candidates. No differences were observed in the binding of wild-type and M77Q calmodulin. Since the arrays were probed with a relatively high concentration of the calmodulins, the method would not detect differences in affinity of the two calmodulin forms. Nevertheless, the failure to identify candidates left unanswered the question of whether M77Q participated in regulatory functions in vivo.

We therefore generated a mutant mouse in which the M77Q calmodulin replaced wild-type calmodulin-1, giving a model of constitutive expression of calmodulin with MetO at residue 77. Learning and cardiac function was normal in the tests that we administered. Phenotypic characterization of the M77Q demonstrated substantive in vivo effects. Their growth, presumably prenatal as well as postnatal, was decreased so that mature M77Q mice weighed 20% less than wild-type littermates but with age they became obese.

The mutant mice were noticeably more active at a young age, a subjective evaluation that was confirmed by objective testing and they ran longer in the forced treadmill test. As noted above, the differences were no longer observed at 10 months of age, perhaps because of increased fat mass. The differences established by neurobehavioral testing resembled those described for knockout mice lacking CaMKIIα. In vitro assays with recombinant proteins established that M77MetO calmodulin was less effective than the wild type in activating recombinant CaMKIIα. Thus, characterization of the phenotype of a mouse expressing a constitutively active mimic of calmodulin oxidized at Met77 led to the identification of the first calmodulin target that is differentially affected by the oxidation state of Met77. The in vivo significance of the difference must be evaluated in future experiments, for example, by determining the fractional phosphorylation of CAMKIIα substrates. Additional studies must also assess which, if any, of the phenotypic changes in the M77Q mouse are a consequence of this differential effect.

## Figures and Tables

**Table 1:** Cardiac parameters from dobutamine stress tests at 6 months of age.

	Volume of LV in Systole (mm^3^)	Volume of LV in Diastole (mm^3^)	Ejection Fraction (%)	Diam LV Chamber in Systole (mm)	Diam LV Chamber in Diastole (mm)	Heart Rate (Beats Per Minute)
Baseline						
WT female	29.2 ± 3.4	72.3 ± 7.4	60 ± 0.8	2.8 ± 0.1	4 ± 0.17	491.9 ± 45.8
M77Q female	30.5 ± 2.2	72.1 ± 5.1	58.9 ± 1.5	2.8 ± 0.1	4 ± 0.12	553.9 ± 34.4
WT male	31 ± 3.1	77 ± 4.7	59.8 ± 2.4	2.9 ± 0.1	4.2 ± 0.1	502.3 ± 33.8
M77Q female	30.78 ± 2.5	78.8 ± 5.5	60.9 ± 1.7	2.8 ± 0.08	4.2 ± 0.12	564.4 ± 18.3
Low Dose						
WT female	16 ± 3.7	59 ± 9.1	73 ± 2.7	2.2 ± 0.2	3.7 ± 0.2	558 ± 40.4
M77Q female	18.1 ± 5.3	60.4 ± 7.4	70.7 ± 5.3	2.3 ± 0.3	3.8 ± 0.2	625.1 ± 18.4
WT male	18.8± 5.1	65.3 ± 7.6	71.7 ± 5	2.3 ± 0.2	3.9 ± 0.2	623.5 ± 37.5
M77Q female	19 ± 8.6	66.7 ± 11.9	73.1 ± 9	2.3 ± 0.5	3.9 ± 0.3	630.6 ± 23.6
High dose						
WT female	13.2 ± 3.5	55 ± 11.5	76.2 ± 3.9	2.0 ± 0.2	3.6 ± 0.3	587.3 ± 47.4
M77Q female	13.2 ± 2.9	57.2 ± 5.7	77.2 ± 4.0	2.0 ± 0.2	3.7 ± 0.2	652.6 ± 21.3
WT male	13.4 ± 2.8	59.4 ± 5.8	77.6 ± 3.8	2.0 ± 0.2	3.7 ± 0.2	667.3 ± 49.2
M77Q female	15.6 ± 7.4	67.3 ± 10.3	77.8 ± 8	2.1 ± 0.4	3.9 ± 0.3	659.4 ± 25.8

## Availability of Calmodulin M77Q Mice

Breeders will be made available to all qualified investigators. Collaboration with the authors is not required.

## References

[1] Levine R.L., Mosoni L., Berlett B.S., Stadtman E.R. (1996). Methionine residues as endogenous antioxidants in proteins. Proc. Natl. Acad. Sci. USA.

[2] Bender A., Hajieva P., Moosmann B. (2008). Adaptive antioxidant methionine accumulation in respiratory chain complexes explains the use of a deviant genetic code in mitochondria. Proc. Natl. Acad. Sci. USA.

[3] Valley C.C., Cembran A., Perlmutter J.D., Lewis A.K., Labello N.P., Gao J., Sachs J.N. (2012). The methionine-aromatic motif plays a unique role in stabilizing protein structure. J. Biol. Chem..

[4] Lee B.C., Peterfi Z., Hoffmann F.W., Moore R.E., Kaya A., Avanesov A., Tarrago L., Zhou Y., Weerapana E., Fomenko D.E. (2013). MsrB1 and MICALs regulate actin assembly and macrophage function via reversible stereoselective methionine oxidation. Mol. Cell.

[5] Hung R.J., Spaeth C.S., Yesilyurt H.G., Terman J.R. (2013). SelR reverses Mical-mediated oxidation of actin to regulate F-actin dynamics. Nat. Cell Biol..

[6] Hung R.-J., Pak C.W., Terman J.R. (2011). Direct redox regulation of F-actin assembly and disassembly by Mical. Science.

[7] Netzer N., Goodenbour J.M., David A., Dittmar K.A., Jones R.B., Schneider J.R., Boone D., Eves E.M., Rosner M.R., Gibbs J.S. (2009). Innate immune and chemically triggered oxidative stress modifies translational fidelity. Nature.

[8] Lee J.Y., Kim D.G., Kim B.G., Yang W.S., Hong J., Kang T., Oh Y.S., Kim K.R., Han B.W., Hwang B.J. (2014). Promiscuous methionyl-tRNA synthetase mediates adaptive mistranslation to protect cells against oxidative stress. J. Cell Sci..

[9] Lim J.C., You Z., Kim G., Levine R.L. (2011). Methionine sulfoxide reductase A is a stereospecific methionine oxidase. Proc. Natl. Acad. Sci. USA.

[10] Lim J.C., Kim G., Levine R.L. (2013). Stereospecific oxidation of calmodulin by methionine sulfoxide reductase A. Free Radic. Biol. Med..

[11] Yap K., Kim J., Truong K., Sherman M., Yuan T., Ikura M. (2000). Calmodulin target database. J. Struct. Funct. Genomics.

[12] Jiang G., Wu F., Li Z., Li T., Gupta V.K., Duan X., Jiang Y. (2018). Sulfoxidation regulation of Musa acuminata calmodulin (MaCaM) Influences the functions of MaCaM-binding proteins. Plant Cell Physiol..

[13] Friedberg F., Rhoads A.R. (2001). Evolutionary aspects of calmodulin. IUBMB Life.

[14] Chin D., Means A.R. (1996). Methionine to glutamine substitutions in the C-terminal domain of calmodulin impair the activation of three protein kinases. J. Biol. Chem..

[15] Drazic A., Miura H., Peschek J., Le Y., Bach N.C., Kriehuber T., Winter J. (2013). Methionine oxidation activates a transcription factor in response to oxidative stress. Proc. Natl. Acad. Sci. USA.

[16] Veredas F.J., Cantón F.R., Aledo J.C. (2017). Methionine residues around phosphorylation sites are preferentially oxidized in vivo under stress conditions. Sci. Rep..

[17] Bigelow D.J., Squier T.C. (2011). Thioredoxin-dependent redox regulation of cellular signaling and stress response through reversible oxidation of methionines. Mol Biosyst.

[18] Bradford M.M. (1976). A rapid and sensitive method for the quantitation of microgram quantities of protein utilizing the principle of protein-dye binding. Anal. Biochem..

[19] National Research Council (2011). Guide for the Care and Use of Laboratory Animals.

[20] CRISPR Design. http://crispr.mit.edu/.

[21] Thermo Fisher Scientific ProtoArray® Applications Guide. http://tools.thermofisher.com/content/sfs/manuals/protoarray_applicationsguide_man.pdf.

[22] Lim J.M., Lim J.C., Kim G., Levine R.L. (2018). Myristoylated methionine sulfoxide reductase A is a late endosomal protein. J. Biol. Chem..

[23] Kregel K.C., Allen D.L., Booth F.W., Fleshner M.R., Henriksen E.J., Musch T., O’Leary D., Parks C., Poole D., Ra’anan A. Resource Book for the Design of Animal Exercise Protocols. http://www.the-aps.org/mm/SciencePolicy/AnimalResearch/Publications/Animal-Exercise-Protocols/book14824.pdf.

[24] Pritchett K., Mulder G.B. (2003). Open-field assessment of spontaneous activity. J. Amer. Assoc. Lab. Animal Sci..

[25] Kim J.J., Jung M.W. (2006). Neural circuits and mechanisms involved in Pavlovian fear conditioning: A critical review. Neurosci. Biobehav. Rev..

[26] Mulder G.B., Pritchett K. (2003). The Morris water maze. J. Amer. Assoc. Lab. Animal Sci..

[27] Mignogna P., Viggiano D. (2010). Brain distribution of genes related to changes in locomotor activity. Physiol. Behav..

[28] Chen C., Rainnie D.G., Greene R.W., Tonegawa S. (1994). Abnormal fear response and aggressive behavior in mutant mice deficient for alpha-calcium-calmodulin kinase II. Science.

[29] Rellos P., Pike A.C.W., Niesen F.H., Salah E., Lee W.H., von Delft F., Knapp S. (2010). Structure of the CaMKIIδ/Calmodulin complex reveals the molecular mechanism of CaMKII kinase activation. PLOS Biol..

[30] Persechini A., Cronk B. (1999). The relationship between the free concentrations of Ca^2+^ and Ca^2+^-calmodulin in intact cells. J. Biol. Chem..

[31] Finn B.E., Forsén S. (1995). The evolving model of calmodulin structure, function and activation. Structure.

